# Understanding and Combating Misinformation: An Evolutionary Perspective

**DOI:** 10.2196/65521

**Published:** 2024-12-27

**Authors:** Nicola Luigi Bragazzi, Sergio Garbarino

**Affiliations:** 1 Division of Human Nutrition Unit Department of Food and Drugs University of Parma Parma Italy; 2 Laboratory for Industrial and Applied Mathematics Department of Mathematics and Statistics York University Toronto, ON Canada; 3 Department of Neuroscience, Rehabilitation, Ophthalmology, Genetics and Maternal/Child Sciences University of Genoa Genoa Italy

**Keywords:** misinformation, infodemics, evolutionary theory, fake news, spoof news, fact-checking, digital platform, behavioral research, social cohesion, extrapolation, deformation, fabrication, disinformation, evolutionary paradox, adaptive qualities, strategic deception, intrapolation, health information, public health

## Abstract

Misinformation represents an evolutionary paradox: despite its harmful impact on society, it persists and evolves, thriving in the information-rich environment of the digital age. This paradox challenges the conventional expectation that detrimental entities should diminish over time. The persistence of misinformation, despite advancements in fact-checking and verification tools, suggests that it possesses adaptive qualities that enable it to survive and propagate. This paper explores how misinformation, as a blend of truth and fiction, continues to resonate with audiences. The role of narratives in human history, particularly in the evolution of Homo narrans, underscores the enduring influence of storytelling on cultural and social cohesion. Despite the increasing ability of individuals to verify the accuracy of sources, misinformation remains a significant challenge, often spreading rapidly through digital platforms. Current behavioral research tends to treat misinformation as completely irrational, static, finite entities that can be definitively debunked, overlooking their dynamic and evolving nature. This approach limits our understanding of the behavioral and societal factors driving the transformation of misinformation over time. The persistence of misinformation can be attributed to several factors, including its role in fostering social cohesion, its perceived short-term benefits, and its use in strategic deception. Techniques such as extrapolation, intrapolation, deformation, cherry-picking, and fabrication contribute to the production and spread of misinformation. Understanding these processes and the evolutionary advantages they confer is crucial for developing effective strategies to counter misinformation. By promoting transparency, critical thinking, and accurate information, society can begin to address the root causes of misinformation and create a more resilient information environment.

## The Evolutionary Paradox of Misinformation

Some stories are unbelievable, yet they can still convince people because, “in substance,” they are true, even though only some details are real and the rest is false. This is the conclusion of “Emma Zunz,” a short story by Argentine writer Jorge Luis Borges (1899-1986) [1], which illustrates how truth and falsehood can blend (“true lies” and “false truths”) [2] and how human life is a complex admixture of fact and fiction. “Emma Zunz” demonstrates how narratives, even when not entirely factual or even completely fabricated, can be perceived as “essentially true,” being powerful, persuasive, and impactful as long as they resonate with the audience [3].

Narratives have played a key role in the story of *Homo narrans* [4] since the earliest days of civilization. From the ancient myths that sought to explain the mysteries of the universe to the epic poems that celebrated heroes and their extraordinary deeds, storytelling has been an integral part of human culture and communication. These narratives not only served to entertain but also to pass down knowledge, moral lessons, and cultural values from one generation to the next. Through storytelling, societies have preserved their histories, shaped their identities, and connected with one another, demonstrating the enduring power of narrative throughout the ages [5].

More specifically, six functions of stories have been proposed: namely, (1) communication, (2) the formation of both individual and collective identities, (3) the cultivation of empathy and theory-of-mind, (4) the development and transmission of knowledge, including social knowledge and tacit sociocultural understanding, (5) the use of stories as tools for simulation and modeling to inform decision-making, and (6) their role in persuasion, particularly in belief change. Notably, the latter 5 functions highlight the role of stories as constitutive forces, shaping realities and understanding, rather than merely reflecting or representing them (constitutive vs representational or descriptive stories) [6].

However, when not completely accurate, some narratives can be detrimental and harmful. As such, the ability of humans to verify the accuracy of sources has evolved and significantly increased over time, especially with the advent of advanced technology and the widespread availability of information. Tools and methods for fact-checking are more accessible than ever, enabling people to cross-reference data, identify misinformation, and scrutinize the credibility of their sources. Despite these advancements, this enhanced capacity for verification has not resulted in the complete elimination or extinction of false news. In fact, misinformation continues to proliferate, often spreading rapidly through digital platforms [7-9], where it can still mislead and influence large audiences. The persistence of false news highlights the ongoing challenges in distinguishing truth from falsehood in the information age, despite the improved tools at our disposal.

However, current behavioral research tends to overlook the dynamic, evolving nature of misinformation, often treating it as an irrational, static, and finite phenomenon. Scholars commonly assume that misinformation is just the product of irrational reasoning, consisting of discrete, fully formed pieces of inconsistent content that can be definitively debunked and falsified. This conventional approach has the advantage of making misinformation more manageable and easier to study in controlled, experimental settings. On the other hand, this irrational, finite perspective comes with a significant drawback: it hinders the comprehension of why it resists scrutiny, obscuring the dynamics and the evolving characteristics of misinformation, and leaving researchers unaware of the behavioral (and societal) factors that drive its transformation over time. As a result, the intricate processes by which misinformation is fabricated, spreads, adapts, and changes are often ignored, limiting our understanding of its impact and persistence in society [10].

Furthermore, misinformation is (apparently) paradoxical from an evolutionary perspective. This paradox lies in the fact that, despite misinformation being harmful and detrimental to society, it persists and continues to spread. According to evolutionary logic, harmful entities should be eliminated or should diminish over time; however, misinformation defies this expectation by thriving and evolving, even in environments where tools and mechanisms exist to detect and remove them. This persistence suggests that misinformation may possess adaptive qualities that enable it to survive and propagate, despite its negative impact on individuals and communities.

Misinformation, along with its counterparts disinformation, malinformation, and deinformation, represents a significant challenge in the modern digital age. Understanding why these forms of information persist and spread requires an examination of their evolutionary advantages, challenging the common views (and prejudices) of misinformation. By analyzing the distinct types of false information and applying an evolutionary framework, we can develop strategies to combat their influence more effectively.

## Misinformation as Units of the Evolutionary Process

As stated by Marchetti and Mastrogiorgio, misinformation “can be considered units of the evolutionary process” [10]. The evolutionary theory, originally formulated by Charles Darwin, has been refined and adapted to explain how cultural information spreads and evolves. In the theory of memes, proposed by Richard Dawkins in 1976 [11], memes are units of cultural transmission or ideas that propagate within a society, analogous to the way genes transmit biological information. Memes can include beliefs, behaviors, symbols, or practices that are passed from one individual to another through communication, imitation, and other forms of social interaction. Much like genes, memes undergo processes of variation, competition, and inheritance, evolving over time based on their ability to replicate and spread. Dawkins’ concept of memes has been influential in fields such as cultural evolution, communication studies, and psychology, offering insights into how cultural phenomena—ranging from language and religion to fashion trends and internet virality—emerge and persist. Adding to the conversation on how information spreads and evolves, Eva Jablonka and Marion Lamb's theory of the “four dimensions of evolution” [12] provides a broader and more nuanced framework for understanding inheritance and evolution, both biological and cultural. Jablonka and Lamb argue that evolution operates on multiple levels, not just through genetic changes but also through other forms of inheritance. They propose 4 dimensions of evolutionary processes: genetic inheritance, epigenetic inheritance, behavioral inheritance, and symbolic inheritance.

Genetic inheritance is the traditional form of inheritance, where traits are passed down through DNA. Beyond DNA, epigenetic factors such as chemical modifications to DNA and histones can influence gene expression and can sometimes be passed on to subsequent generations. This form of inheritance allows for environmental factors to affect evolutionary outcomes, offering a more dynamic view of how organisms adapt and evolve. Behavioral inheritance is a dimension including behaviors that are learned and transmitted across generations (for example, animals teaching their offspring how to hunt or humans passing down cultural practices). Behavioral inheritance is crucial in the context of cultural evolution, where learned behaviors can have significant adaptive value. The fourth dimension, symbolic inheritance, refers to the transmission of information through symbols, language, and other forms of communication unique to humans. Symbolic inheritance encompasses the spread of memes and is essential for understanding how complex cultural systems—such as religious beliefs, scientific knowledge, and social norms—develop and persist.

By integrating these 4 dimensions, Jablonka and Lamb’s theory offers a comprehensive view of how both biological and cultural evolution are driven by a variety of mechanisms. It also highlights the interconnectedness of these processes, where cultural practices can influence genetic evolution, and vice versa.

In the context of misinformation and cultural transmission, both meme theory and the 4 dimensions of evolution can provide valuable insights. Misinformation can be understood as a type of meme that evolves within the symbolic inheritance system, spreading through communication and social media. Their persistence and adaptation over time can be analyzed through the lens of behavioral inheritance, as individuals and groups learn to create, share, and reinforce these narratives. The interaction between genetic, epigenetic, behavioral, and symbolic dimensions could also explain why some individuals are more prone to misinformation and why certain types of misinformation are more resilient and pervasive than others, pointing to the complex interplay between different forms of inheritance, evolution, and behaviors.

## The Distinction Among Misinformation, Disinformation, Malinformation, and Deinformation

According to the literature, the various forms of misleading information can be categorized as follows: deinformation, malinformation, misinformation, and disinformation [10,13,14]. Based on the “ABCDE framework,” they vary according to the actors disseminating misinformation (A), their intentions and behaviors (B), the content disseminated and its context (C), the degree of its distribution in terms of audience reached (D), and the effect or impact (harm or threat) caused or potential (E) [15-17].

Deinformation is a less commonly discussed category where specific content is true but the overall message becomes unintentionally false due to a lack of context or competence by the producer. It can be seen as an unintentional form of sensationalism. Malinformation involves the use of true information with harmful intent. While the facts themselves are accurate, they are presented or highlighted in a way designed to cause harm, often through mechanisms like cherry-picking or sensationalism. An example of malinformation is doxing, which consists in the public disclosing and sharing of private information. Misinformation refers to false information that is created without harmful intent. It is often spread by individuals who believe the information to be true, making it a “benign” form that resembles traditional news in its presentation. Disinformation is false information deliberately created with the intent to deceive or cause harm. This is the most insidious type, as it is crafted with malicious purposes, such as manipulating public opinion or discrediting individuals or groups.

These categories help differentiate the various ways in which information can be manipulated or fabricated. Recognizing these distinctions is crucial for developing tailored strategies to counter each type effectively.

## Information Along the Truth-Falsehood Continuum

The “deinformation / malinformation / misinformation / disinformation” categories can be thought of along a truth-falsehood *continuum*, where each type of information occupies a different position based on its degree of truthfulness, accuracy, and the intent behind its creation and dissemination.

At one end of the *spectrum*, we have deinformation, which starts with true content but becomes misleading due to a lack of context or competence, resulting in an unintentionally false overall message. This reflects low intent to deceive but still carries a risk of causing misunderstanding. Moving further along the *continuum*, malinformation represents accurate information used with malicious intent. Here, the truthfulness of the content is high, but the intent to harm, deceive, or manipulate is also significant, often leading to negative consequences despite the factual nature of the information. Misinformation, positioned closer to the middle of the *continuum*, involves false information shared without the intent to deceive. The accuracy of the content is low, but the intent behind its dissemination is typically benign, as the individuals spreading it usually believe it to be true. At the opposite end of the *spectrum* lies disinformation, which is entirely false and created with a clear intent to deceive, manipulate, or harm. This represents the most dangerous category, combining low accuracy with high malicious intent, resulting in significant potential for harmful impact.

Together, these categories illustrate how information can vary not only in terms of its factual correctness but also in the motivations behind its creation and the potential harm it can cause. Understanding where a piece of information falls on this *continuum* can help in assessing its reliability and the potential risks associated with its dissemination (Table 1).

**Table 1:** Information and techniques used to produce and deliver information along the “truth-falsehood continuum,” categorized by accuracy, intent, and potential harm.

Category and technique		Description	Accuracy	Intent	Potential harm
**Deinformation**					
	Extrapolation	Extending true data points beyond their original context to draw broader, often misleading, conclusions	High (initial facts)	Low	Moderate (misleading message)
	Intrapolation	Inserting false or misleading details within a true context, distorting the original message	Mixed (true context, false details)	Low	Moderate
**Malinformation**					
	Cherry-picking	Selecting specific pieces of true information to support a viewpoint while ignoring contradictory evidence	High (selected facts)	High	High (intentional omission)
	Contextualization	Placing true information within a misleading context to alter its interpretation	High (facts)	High	High (manipulative context)
	Misrepresentation	Presenting information in a way that unintentionally misleads, often by altering context or tone	Low (false presentation)	Low	Moderate (unintentional)
**Disinformation**					
	Deformation	Altering facts or details to fit a specific narrative, often by twisting or exaggerating the truth	Low (twisted facts)	High	Very high (intentional)
	Fabrication	Creating completely false information or events with no basis in reality	None (false information)	High	Very high (intentional)
	Echo chamber amplification	Repeating or amplifying false or misleading information within a closed group, reinforcing the false narrative	None or low (false information)	High	Very high (reinforced belief)

## The Role of Falsehood in Contributing to Knowledge

Paradoxically, falsehood can contribute to knowledge. As stated by Bernecker [18], “falsehood plays an important role in the inference-based production of knowledge” by serving as a catalyst for clarifying concepts, strengthening arguments, and enhancing understanding. When engaging with falsehoods, thinkers often test assumptions and explore alternative possibilities that might initially seem plausible. This process helps refine and redefine the boundaries of true knowledge.

By critically examining and refuting false ideas, the reasoning process is sharpened, and the credibility of true knowledge is bolstered. In philosophical traditions, particularly in the dialectical method, falsehoods are often introduced deliberately to stimulate discussion and challenge existing beliefs. This confrontation between opposing ideas leads to a synthesis that represents a more refined understanding. In education, falsehoods are also used as a pedagogical tool to provoke critical thinking. When students are challenged with false statements, they must apply their knowledge to refute them, which reinforces their learning and deepens their comprehension.

The role of falsehood in scientific progress is particularly notable in the context of Karl Popper’s falsifiability (or refutability) principle [19]. Scientific knowledge advances through conjectures and refutations, where theories must be falsifiable. The process of attempting to falsify a theory, and encountering falsehoods along the way, is crucial for scientific advancement. When a theory withstands these challenges, our confidence in its truth increases.

The discovery and correction of errors within scientific theories also contribute to the self-correcting nature of science, driving it closer to the truth. In hypothetical reasoning, falsehoods are used in counterfactual scenarios, which, although not true, provide valuable insights into causal relationships and decision-making processes. These scenarios help in understanding the underlying principles of the subject matter. Furthermore, falsehoods help identify exceptions to general rules and clarify the limitations of existing knowledge. By recognizing where and why certain inferences do not hold, theories can be refined to accommodate a broader range of phenomena.

In sum, falsehoods are essential in the production of knowledge through inference. They challenge existing beliefs, prompt critical examination, and drive the process of inquiry and discovery, ultimately leading to a more robust and nuanced understanding of the world.

## Falsehood as Non-(Completely) Irrational

False beliefs and beliefs in conspiracy theories are generally considered inherently irrational due to their resistance to disconfirming evidence and internal incoherence. However, Poth and Dolega [20] argued that such beliefs can be considered rational within a probabilistic framework. More in detail, belief in conspiracy theories can be rational if it is supported by a network of auxiliary beliefs that protect the core belief from disconfirmation. Within this network of beliefs, auxiliary hypotheses are adjusted to protect a core hypothesis from falsification. Central conspiracy beliefs can be, indeed, preserved by revising or rejecting auxiliary beliefs, which may be more easily discarded when faced with disconfirming evidence. This process aligns with Bayesian norms of rationality, where the updating of beliefs depends on prior probabilities and the likelihood of new evidence. The monological nature of conspiracy beliefs (where beliefs support each other in a self-sustaining network) can be reconciled with their apparent insensitivity to counterevidence by viewing such beliefs as different aspects of the same phenomenon within a Bayesian framework. Inductive biases have a role in the formation and maintenance of conspiracy beliefs, as they influence how individuals weigh the evidence and update their beliefs, potentially leading to the adoption of conspiracy theories even in the face of contradictory evidence. Finally, a distinction should be made between rationality and desperation, between “glorious rescues” of beliefs (where adjustments lead to new, confirmable predictions) and “desperate rescues” (where auxiliary beliefs are adjusted without sufficient justification). The latter is considered a hallmark of irrational belief maintenance. In conclusion, belief in conspiracy theories can be rational in certain contexts, especially when viewed through a Bayesian lens. However, the rationality of these beliefs depends on the broader context, including the strength of prior beliefs and the nature of the evidence encountered. The irrationality of some conspiracy beliefs may stem more from desperate attempts to protect poorly confirmed hypotheses rather than a fundamental flaw in the belief-updating process itself.

## Techniques of Misinformation Production

Several techniques can be employed in the production of misinformation, including (1) extrapolation, (2) intrapolation, (3) deformation, (4) cherry-picking, (5) misrepresentation, (6) contextualization, (7) fabrication, and (8) echo chamber amplification. Each of these methods plays a distinct role in distorting, manipulating, and fabricating information to create misleading narratives.

Extrapolation involves taking specific facts or data points and extending them beyond their original context to draw broader, often misleading, conclusions. Intrapolation refers to manipulating information within its context, often by inserting false or misleading details that distort the original message while retaining a semblance of truth. Deformation is altering facts or details to create a narrative that fits specific purposes, such as a particular agenda, often by twisting or exaggerating the truth. Cherry-picking refers to selecting only specific pieces of information that support a particular viewpoint or narrative while ignoring contradictory evidence. Misrepresentation involves presenting information in a way that intentionally misleads, often by changing the context, tone, or framing of the content. Contextualization refers to placing true information within a misleading context to alter its interpretation, often to provoke a particular reaction or belief. Fabrication is the creation of completely false information or events that are presented as truth, with no basis in reality. Finally, echo chamber amplification involves repeating or amplifying false or misleading information within a closed or like-minded group, reinforcing the narrative and making it seem more credible.

## Becoming True and Becoming False Versus Being True and Being False

True and false news are not merely static collections of facts; they are dynamic processes [10]. The information we encounter often goes through various stages of verification, interpretation, and dissemination, which can affect its status as true or false. True news is not simply about reporting facts accurately. It involves a rigorous process of gathering, verifying, and contextualizing information. This process includes cross-checking sources, analyzing data, and presenting the information in a way that aligns with objective reality. As new information becomes available, what was once thought to be true can be refined or even corrected, highlighting the ongoing nature of truth. Similarly, false news is not just the presentation of incorrect information; it often involves deliberate or unintentional processes that distort or misrepresent facts. This could include selective reporting, biased framing, or the spread of unverified information. Over time, as this false information circulates, it can be amplified and accepted by some as true, further complicating the distinction between truth and falsehood.

News, whether true or false, is often in a state of “becoming.” A story may begin as a rumor, undergo various degrees of scrutiny, and eventually be confirmed as true or debunked as false. The “becoming” aspect emphasizes that the truthfulness of news is not always immediate or self-evident. It is something that can evolve, depending on how the information is handled, interpreted, and understood by both journalists and the public. In this way, true and false news are processes that involve ongoing evaluation and re-evaluation, making them dynamic rather than fixed entities.

## From False Claims and Statements to False Narratives, Conspiracy Theories, and False Belief Systems

The journey from false claims and statements to the development of false narratives, conspiracy theories [21,22], and false belief systems [23] is a complex and often insidious process. It begins with a false claim, which is simply an assertion that is factually incorrect. These false claims can be made either deliberately, as lies, or accidentally, as misinformation. If these claims go unchecked, they can spread, leading people to accept these falsehoods as truth. When multiple false claims are strung together, they can form what is known as a false narrative. A false narrative is more than just a collection of incorrect statements; it is a story or explanation that weaves these claims into a broader context that appears coherent and credible. These narratives often simplify complex situations or manipulate facts to fit a particular agenda, making them persuasive to those who encounter them. The impact of false narratives can be significant, influencing public opinion, driving decision-making, and justifying actions based on incorrect or incomplete information. As false narratives gain traction, they can evolve into conspiracy theories. A conspiracy theory is a specific type of false narrative that suggests a secret, often sinister, plot by a group of people or organizations. These theories are typically based on speculation and lack solid evidence, yet they thrive on the distrust of official explanations or the desire to explain complex phenomena in emotionally charged, simplistic ways. The danger of conspiracy theories lies in their ability to challenge established facts, foster distrust in institutions, create social division or even incite violence. Over time, these narratives and conspiracy theories can harden into false belief systems. A false belief system is a cohesive set of beliefs that are based on incorrect information or flawed reasoning. These systems can be religious, political, or ideological in nature and are often deeply ingrained in the individuals or groups that hold them. As people become increasingly committed to these beliefs, they become resistant to contrary evidence, making these belief systems difficult to challenge. The impact of false belief systems is profound, as they can shape individual and collective identities, influence behavior, and perpetuate misinformation across generations. The progression from false claims to false belief systems often follows a recognizable path: false claims give rise to false narratives, which can then develop into conspiracy theories, and ultimately solidify into false belief systems. This progression can be seen in various real-world examples, such as the Flat Earth theory, which began as a false claim, evolved into a narrative questioning mainstream science, became a conspiracy theory involving governments and scientists, and now exists as a false belief system with a community of believers. Similarly, the antivaccine movement started with false claims about the dangers of vaccines, developed into a narrative suggesting vaccines are part of a harmful plot, led to conspiracy theories about government and pharmaceutical companies, and has now become a widespread false belief system influencing public health [24].

## Evolutionary Advantages of Misinformation

From an evolutionary perspective, the prevalence of misinformation over correct information can be understood through the lens of how human cognition and social behavior have evolved: (1) survival value of heuristics, (2) social cohesion and group identity, (3) emotional resonance, (4) information overload and cognitive limits, (5) status and influence, and (6) mimicry and the spread of beliefs.

Throughout human evolution, our ancestors relied on mental shortcuts, or heuristics, to make quick decisions in uncertain environments. These heuristics were often based on incomplete or ambiguous information but were crucial for survival. For example, reacting quickly to a potential threat based on limited information (eg, rustling in the bushes might be a predator) was more important than verifying the accuracy of the threat. This predisposition to favor quick, heuristic-based decisions over slow, analytical reasoning persists in modern humans, making us susceptible to misinformation that triggers these quick judgments.

Humans are inherently social animals, and our evolutionary success has been closely tied to our ability to form cohesive groups. Sharing and believing in the same information (even if incorrect) can strengthen group identity and solidarity. Misinformation that aligns with group norms or beliefs may be more readily accepted because it reinforces social bonds. This can be especially powerful in times of uncertainty or conflict, where group cohesion is critical for survival.

Evolutionarily, emotions have played a critical role in decision-making. Information that elicits strong emotions—fear, anger, or excitement—can trigger faster and more decisive actions, which would have been advantageous in survival contexts. Misinformation often spreads because it is emotionally charged, capturing attention and encouraging rapid dissemination, much like how a warning about a potential danger would have spread quickly in early human communities.

The human brain evolved to process a manageable amount of information in relatively stable environments. In today's world; however, we are bombarded with vast amounts of information daily. Evolutionarily, we are not well-equipped to handle this overload, leading to reliance on simple narratives, even if they are inaccurate. Misinformation often provides these simple, digestible narratives that our brains prefer, especially under conditions of information overload. Moreover, storytelling has been a fundamental way humans have passed down knowledge and information through generations. Evolutionarily, stories that were memorable, engaging, and had clear moral lessons were more likely to be retained and shared. Misinformation often comes in the form of compelling stories that are easier to remember and share, even if they are not true.

In ancestral human societies, individuals who were able to provide information (whether true or not) that influenced group decisions could gain status and power within the group. This dynamic still exists today, where spreading sensational or shocking information can elevate a person's status, especially on social media. The desire for social status can drive the spread of misinformation as individuals seek to gain attention and influence.

From an evolutionary standpoint, humans have developed a tendency to imitate the behavior and beliefs of those around them, especially individuals who are perceived as successful or influential. This mimicry would have been beneficial in many survival contexts but can also lead to the spread of misinformation when influential figures or a majority within a group propagate false information.

In summary, from an evolutionary perspective, the factors that once helped our ancestors survive—such as quick decision-making, social cohesion, emotional responsiveness, and storytelling—can also make modern humans more susceptible to misinformation. These deep-rooted tendencies, shaped by millennia of evolution, create fertile ground for misinformation to thrive in contemporary society.

## Combating Misinformation

Appreciating the dynamics and evolutionary trajectories of misinformation is key to combating misinformation.

As previously said, misinformation can play a key role in fostering social cohesion and reinforcing group identity. Shared beliefs, even if inaccurate, can unite communities, creating a strong sense of belonging. This dynamic is evident in how myths, conspiracy theories, and certain forms of disinformation become rallying points for groups, strengthening their internal bonds while distinguishing them from outsiders. To counteract this, it is essential to offer alternative narratives that promote unity without relying on falsehoods. Encouraging communities to embrace values grounded in truth can weaken the social cohesion that misinformation creates. Moreover, fostering inclusivity within communities can reduce the appeal of misinformation-driven identities, promoting dialogue and understanding across social divides.

From an evolutionary standpoint, misinformation may sometimes appear to offer survival or reproductive benefits. For instance, deceptive signals or behaviors can deter competitors or attract mates, providing immediate, though misleading, advantages. However, the long-term risks associated with misinformation—such as poor decision-making or health consequences—often outweigh these short-term gains. By highlighting these risks, individuals can be made aware of the dangers inherent in clinging to false beliefs. Education on the value of accurate information, particularly in matters of health and safety, can help individuals recognize the limitations and potential harms of relying on misinformation.

Misinformation is frequently employed as a tool for strategic deception in various contexts, including social, political, and economic arenas. To mitigate this, promoting transparency and accountability is critical. If the benefits of deception are reduced, misinformation becomes a less attractive option. Strengthening the role of fact-checking organizations and technologies that can quickly identify and correct false information can also diminish the effectiveness of misinformation. When individuals and organizations face consequences for spreading misinformation, the incentive to engage in such practices diminishes.

In complex and uncertain environments, misinformation often provides simplified narratives that help individuals make quick decisions, even if those decisions are based on falsehoods. Combating this requires the provision of clear, concise, and easily digestible, accurate information. Simplified yet truthful narratives can compete with misinformation by reducing the cognitive burden required to understand complex topics. Additionally, ensuring that accurate information is available and disseminated quickly, particularly during crises, limits the space for misinformation to take hold. The rapid presentation of truth can preempt the spread of falsehoods that thrive in the vacuum of uncertainty.

Certain forms of misinformation persist because they can indirectly reinforce socially desirable behaviors, even if the factual basis is incorrect. These false beliefs may be rooted in myths, taboos, or cultural narratives that promote actions beneficial to the community. For example, myths about the dangers of overusing certain natural resources might discourage exploitation, thereby preserving the environment. Similarly, health-related taboos, even when scientifically unfounded, may encourage behaviors that reduce the spread of diseases or promote social harmony. In this way, misinformation can be sustained because it serves a protective or stabilizing function for the group, even when the facts themselves are erroneous. Combatting this requires redirecting these motivations toward truthful narratives that also promote social good. By framing accurate information in a way that appeals to prosocial motivations, communities can maintain the positive functions of these beliefs without relying on misinformation. Creating new cultural narratives grounded in factual information can serve as effective replacements for false beliefs, preserving their social benefits while ensuring they are based on reality.

Misinformation can sometimes act as a catalyst for exploration and innovation by sparking curiosity about the unknown. When people encounter mysterious or unverified claims, it can inspire them to investigate further, seeking answers that might reveal new insights or technological advancements. For instance, historical misconceptions about the natural world have occasionally prompted scientific inquiry, leading to groundbreaking discoveries. However, for this curiosity to be productive, it must be guided by critical thinking and skepticism. Without these tools, the exploration may result in reinforcing false beliefs rather than uncovering truths, making it crucial to question and verify the information during the investigative process. As such, fostering a culture of critical thinking, where individuals are encouraged to question and verify information before accepting it, is crucial in combating misinformation. Moreover, education systems should be adaptable, teaching students how to think critically about information sources and adjust their understanding as new, accurate information becomes available. This approach helps counter the flexibility of misinformation, ensuring that innovation and exploration are informed by facts.

## The Taxonomy of Strategies to Tackle Misinformation

There exist several strategies to tackle and combat misinformation [25-28]. These various techniques can be broadly categorized into informational, educational, cognitive, social and community-based, technology-driven, and institutional approaches. Moreover, they can be classified into proactive versus reactive and user-centered versus producer-centered countermisinformation interventions.

Cognitive approaches focus on preparing individuals mentally to resist misinformation. Cognitive inoculation [29], for example, exposes people to weakened forms of misinformation, helping them build resistance when they encounter stronger versions. Similarly, cognitive reflection tasks encourage critical thinking, prompting individuals to reflect before accepting information at face value [30].

Educational approaches aim to empower individuals with the skills and knowledge needed to recognize and reject misinformation. Media and social media literacy education teaches critical evaluation of information sources through classroom-based coursework, short web-based videos, or games, enabling people (especially adolescents and young adults) to identify misinformation [31,32]. Debunking involves directly correcting false claims with evidence-based information, while prebunking preemptively exposes individuals to misinformation tactics, equipping them to spot and resist these tactics in the future [33]. Gamification and interactive tools also fall into this category, using engaging, game-like elements to teach users how to recognize and combat misinformation [34].

Social and community-based approaches leverage the influence of social norms and trusted voices within communities. Social norms messaging works by communicating what behaviors or beliefs are typical or acceptable within a group, encouraging individuals to reject misinformation [35]. Community engagement and peer influence involve enlisting trusted community members to spread accurate information and counter misinformation, relying on the power of peer influence [36].

Technology-driven approaches use digital tools and algorithms to combat misinformation. Algorithmic interventions, for instance, detect and limit the spread of false information on online platforms by demoting or flagging misleading content [37].

When considering the timing and nature of these interventions, they can be further divided into proactive and reactive approaches. Proactive interventions aim to prepare individuals before they encounter misinformation. Cognitive inoculation and prebunking are examples of proactive strategies that build resistance to misinformation in advance. Media literacy education and gamification also serve as proactive tools by equipping individuals with the knowledge and skills to recognize and resist misinformation from the outset.

On the other hand, reactive interventions respond to misinformation after it has been encountered. Fact-checking verifies specific claims and provides corrections after misinformation has been spread, while debunking directly addresses and refutes false information. Algorithmic interventions react to misinformation in real-time by limiting its spread on digital platforms. Narrative correction provides an alternative, truthful narrative to counter misinformation that has already been disseminated.

This classification highlights the diverse strategies employed to combat misinformation, encompassing individual cognitive strategies, community engagement, educational efforts, and technological solutions, and distinguishing between proactive prevention and reactive correction (Table 2).

**Table 2:** Strategies for combating misinformation.

Technique	Description	Application
Cognitive inoculation	Exposing individuals to weakened forms of misinformation to build resistance	Used in public health campaigns to build resistance against persuasive misinformation
Fact-checking	Verifying specific claims and correcting inaccuracies in public discourse	Employed by organizations or fact-checking sites and reference sources to assess the veracity of claims
Media literacy education	Teaching critical evaluation of information sources to recognize misinformation	Implemented in schools, universities, and online platforms to teach media literacy
Debunking	Directly refuting false claims with evidence-based corrections	Common in journalism and science communication to correct false claims
Prebunking	Exposing misinformation tactics to help individuals spot and resist false information	Used by educational campaigns to inform the public about misinformation tactics
Social norms messaging	Communicating what behaviors or beliefs are typical to encourage rejection of misinformation	Applied in public health to encourage following scientific advice by showing majority behavior
Algorithmic interventions	Using algorithms to detect and limit the spread of misinformation online	Implemented by platforms like Facebook, Twitter, and Google to reduce visibility of false content
Community engagement and peer influence	Engaging communities and leveraging trusted voices to counter misinformation	Used in public health and community campaigns to spread accurate information
Narrative correction	Countering misinformation by telling a compelling and truthful story	Used in documentaries and social media to correct widespread myths
Cognitive reflection tasks	Prompting individuals to think critically about information before accepting it	Integrated into educational tools and quizzes that encourage critical thinking
Gamification and interactive tools	Using game-like elements to teach users how to recognize and resist misinformation	Examples include online games that teach users about misinformation tactics

## Tackling Misinformation in a Dynamically Evolving Landscape

As stated by Bateman and Jackson [25], “there is no silver bullet or “best” policy option.” Indeed, none of the interventions previously mentioned are both well-researched, highly effective, and easy to scale at the same time. Instead, the effectiveness of most interventions appears to be quite uncertain and likely hinges on a variety of factors that researchers have only just started to explore.

To effectively implement countermisinformation strategies in a dynamic landscape where false news and beliefs are continuously adapting, a multifaceted approach that evolves alongside the misinformation itself is essential.

One critical aspect is the development of adaptive and continuous learning systems. Technology-driven strategies should include algorithms capable of learning from new patterns of misinformation. By updating machine learning models continuously, these algorithms can detect emerging forms of misinformation through the analysis of content trends, linguistic shifts, and changes in misinformation sources. Platforms can then use these adaptive algorithms not only to flag or demote misleading content but also to adjust their detection criteria as misinformation evolves. Feedback loops are crucial in this context; interventions must be regularly assessed for their effectiveness. If a particular fact-checking or debunking strategy proves less effective over time, adjustments can be made based on user feedback, ensuring that the response to misinformation remains robust. The synergy between proactive and reactive approaches also plays a vital role. Proactive strategies, such as cognitive inoculation and prebunking, need to be regularly updated with the latest misinformation tactics and narratives. This ensures that individuals are prepared to recognize the most recent threats.

On the reactive side, interventions must be agile and quick to deploy. Timely fact-checking or narrative correction should match the speed at which misinformation spreads. This necessitates close collaboration between fact-checkers, social media platforms, and news outlets to ensure a rapid response. Community-driven and peer-influence models offer another layer of defense against misinformation. Engaging community leaders and influencers who can quickly adapt their messaging to counter new forms of misinformation is crucial. These trusted voices can amplify accurate information and address specific community concerns as they arise. Peer-led initiatives can further enhance this effort by encouraging individuals within communities to actively participate in identifying and countering misinformation. Establishing peer-to-peer fact-checking networks can help people share verified information and challenge false claims within their social circles. Continuous education and literacy development are essential components of this adaptive strategy. Media and social media literacy programs should be dynamic, incorporating new case studies and evolving threats. Regular updates to educational content ensure that individuals stay informed about the latest misinformation tactics and how to resist them. Gamified learning tools should also be kept current, with scenarios that reflect the most recent forms of misinformation, maintaining their relevance and effectiveness. A cross-platform and multichannel approach is necessary to address the spread of misinformation across different platforms. Since misinformation spreads differently on various platforms, interventions must be tailored to the specific characteristics of each one. For instance, the tactics required to counter misinformation on Twitter may differ from those on TikTok.

Additionally, a multi-channel communication strategy that includes social media, traditional media, and direct community engagement ensures that accurate information reaches a wide audience, regardless of their preferred information sources. Building resilience through cognitive approaches is another key strategy. Encouraging regular cognitive exercises, such as reflection tasks, helps individuals maintain and enhance their resilience to misinformation over time. Exposure to a wide range of information sources also fosters cognitive flexibility, which is crucial for resisting misinformation. Institutional and policy support further strengthens these efforts. Regulatory frameworks should be flexible and capable of adapting as misinformation evolves. Governments and institutions must collaborate with researchers and technology companies to refine these regulations in response to new challenges. Public-private partnerships can also play a significant role by facilitating the sharing of data, insights, and resources, leading to more coordinated and effective responses to evolving misinformation.

Ongoing research and development are indispensable for staying ahead of misinformation. Continuous research into the dynamics of misinformation—how it evolves, spreads, and impacts different populations—can inform the development of new strategies. Piloting new interventions in controlled environments before scaling them up ensures that these strategies are both effective and scalable.

By integrating these approaches, counter-misinformation efforts can remain effective in a rapidly changing information environment, ensuring that interventions are both proactive and reactive and continuously adapt to the evolving nature of false news and beliefs.

## Conclusions

Understanding the dynamics and the evolutionary advantages of misinformation provides valuable insights into how it can be effectively countered. By recognizing the distinct types of misleading information—misinformation, disinformation, malinformation, and deinformation—as non-completely irrational, dynamically evolving entities, we can tailor strategies to address each form. Promoting transparency, simplifying accurate information, replacing false beliefs with truthful narratives, and encouraging critical thinking are essential components of this effort. By addressing the root causes of misinformation and offering viable alternatives, we can create a more resilient information environment where truth prevails over falsehood.
